# Managing Energy Consumption of Devices with Multiconnectivity by Deep Learning and Software-Defined Networking

**DOI:** 10.3390/s23187699

**Published:** 2023-09-06

**Authors:** Ramiza Shams, Atef Abdrabou, Mohammad Al Bataineh, Kamarul Ariffin Noordin

**Affiliations:** 1 Department of Electrical and Communication Engineering, College of Engineering, United Arab Emirates University, Al-Ain P.O. Box 15551, Abu Dhabi, United Arab Emirates; 201970038@uaeu.ac.ae (R.S.); mffbataineh@uaeu.ac.ae (M.A.B.); 2 Telecommunications Engineering Department, Yarmouk University, Irbid 21163, Jordan; 3 Department of Electrical Engineering, Faculty of Engineering, University of Malaya, Kuala Lumpur 50603, Malaysia; kamarul@um.edu.my

**Keywords:** multipath TCP, software-defined networking, energy consumption, neural networks, congestion control, wireless, multihoming, multiconnectivity

## Abstract

Multiconnectivity allows user equipment/devices to connect to multiple radio access technologies simultaneously, including 5G, 4G (LTE), and WiFi. It is a necessity in meeting the increasing demand for mobile network services for the 5G and beyond wireless networks, while ensuring that mobile operators can still reap the benefits of their present investments. Multipath TCP (MPTCP) has been introduced to allow uninterrupted reliable data transmission over multiconnectivity links. However, energy consumption is a significant issue for multihomed wireless devices since most of them are battery-powered. This paper employs software-defined networking (SDN) and deep neural networks (DNNs) to manage the energy consumption of devices with multiconnectivity running MPTCP. The proposed method involves two lightweight algorithms implemented on an SDN controller, using a real hardware testbed of dual-homed wireless nodes connected to WiFi and cellular networks. The first algorithm determines whether a node should connect to a specific network or both networks. The second algorithm improves the selection made by the first by using a DNN trained on different scenarios, such as various network sizes and MPTCP congestion control algorithms. The results of our extensive experimentation show that this approach effectively reduces energy consumption while providing better network throughput performance compared to using single-path TCP or MPTCP Cubic or BALIA for all nodes.

## 1. Introduction

The massive increase in the data traffic volume and the user demand for high-speed data rates and high-quality services has created a requirement for innovative and adaptable network implementations in the existing wireless technologies. The machine-to-machine (M2M) and the Internet of Things (IoT) paradigms [1] presume the connectivity of billions of wireless devices in diverse application domains, such as smart grids, vehicular networks, and many others, which demands low latency, high reliability, and high throughput.

The fifth-generation (5G) wireless network standard came with radio enhancements (new radio) to provide users/devices with communication services such as ultra-reliable and low-latency (URLLC) communication, high-throughput mobile broadband (eMBB), and the massive support of machine-type communication (mMTC). However, the increasing demand for wireless bandwidth may negatively impact the performance of these services. Thus, the 3GPP introduced a method known as dual connectivity/multiconnectivity, in which a user device can send and receive data concurrently to and from two or multiple nodes using the same (e.g., macro-cell and small cells) or different technologies (e.g., cellular base stations and WiFi access points) [2,3]. This can boost the amount of bandwidth available to a user’s device, help to manage its mobility more effectively, and increase reliability.

At the same time, the emergence of the multipath transmission control protocol (MPTCP) [4] can aid the realization of multiconnectivity by allowing a user to simultaneously transmit through multiple radio interfaces, such as WiFi and LTE or 5G, while providing the reliability of single-path TCP. MPTCP provides concurrent and uninterrupted data transmissions through multiple paths via multiple subflows. Each one is connected to one wireless network. It overcomes the limitations in the traditional TCP by maximizing throughput and redundancy, in addition to providing a resilient reaction to failure [5].

Using devices with dual or multiconnectivity can be challenging due to energy consumption, especially with battery-powered wireless devices used in emerging technologies like IoT. It is crucial for wireless devices to be energy-efficient while providing optimal performance. Higher energy consumption leads to increased battery capacity and a larger device size and weight. Therefore, minimizing energy consumption is crucial to extend the battery life (for the same form factor) and maintain a small carbon footprint. When using two wireless interfaces simultaneously, it is essential to ensure that the overall energy consumed by both interfaces does not significantly exceed that of a single interface.

The operation of the MPTCP protocol relies on two main components, namely a scheduler and a congestion control algorithm. The scheduler selects the traffic path (the wireless interface) for each packet, whereas the congestion control algorithm estimates the amount of data to be sent over each path in order to avoid congestion. The execution of both components controls the traffic share of each interface, which directly affects the energy consumed by each interface, as the more data sent, the longer the time that the interface will be used and hence more energy is consumed. For the first component (the scheduler), the MPTCP Linux kernel implementation uses the default scheduler, which splits the traffic on each path based on the round-trip time (RTT) experienced over that path. However, for the second component (the congestion control algorithm), there are five different algorithms available as Linux kernel implementations, namely OLIA, LIA, wVegas, Cubic, and BALIA [6,7,8,9,10]. To transfer some amount of data (e.g., a file), each algorithm assigns the traffic load differently to the operating wireless interfaces (i.e., LTE/5G and WiFi) and hence it achieves different MPTCP throughput performance and, in turn, consumes a different amount of energy. Thus, this research first experimentally analyzes and compares the energy consumption of devices running MPTCP using the aforementioned congestion control algorithms available as Linux kernel implementations. Since the MPTCP scheduler and congestion control algorithm are the main contributors to the MPTCP operation, various researchers propose modifying the implementation of one, such as in [11,12], or both of them, as in [13,14], to reduce the energy consumption of MPTCP. However, the proposed modification may impact the throughput performance compared to other congestion control algorithms and affect the compatibility with other nodes operating the Linux kernel implementation of single-path TCP or MPTCP.

Therefore, this paper proposes the usage of SDN accompanied by a deep neural network (DNN) to reduce the energy consumption for devices with dual or multiconnectivity. SDN [15] is an evolutionary paradigm with great potential in controlling network traffic flows. It has recently enabled a significant leap forward in the networking industry as it allows networks to become faster, more flexible, automated, and less complicated [16]. In this paradigm, the network data plane and control plane are physically separated, where the control plane manages several devices. The SDN controller generates the forwarding tables and pushes them into the data plane [17]. It provides the necessary commands to configure the network based on an overall network view and software programs written over the controller. Moreover, it paves the way for the control of the network by software functions from a central controller, by avoiding the difficulty of the manual reconfiguration of network devices [18]. The integration of SDN with MPTCP can minimize the energy consumption of wireless devices without modifying the MPTCP implementation [19].

Thus, we propose a scheme employing the SDN concept to control the traffic flows received by each node wireless network interface to minimize the downlink energy consumption. In this scheme, the SDN controller decides on steering the flow of data packets to the receiving nodes based on a lightweight interface selection algorithm relying on the measured achievable throughputs of each node’s wireless interfaces. Moreover, the proposed scheme offers another algorithm that also runs on the SDN controller to fine-tune its interface selection based on energy consumption information provided by a trained DNN.

This paper offers two main contributions. Firstly, it provides a comparative experimental study of the data transfer throughput and energy consumption of different MPTCP congestion control algorithms, namely LIA, OLIA, BALIA, Cubic, and wVegas, which are all available as Linux kernel implementations. Secondly, it presents two algorithms that select which wireless interface should operate in each network node to minimize the overall network energy consumption. These algorithms are implemented using a Ryu-based SDN controller, which manages an Open vSwitch-based switch via the OpenFlow protocol. Based on the comparative study results, the first algorithm selects the operating radio interfaces of each node mainly based on their probed achievable throughput. The second algorithm uses a trained DNN to make an informed decision about whether the interfaces selected by the first algorithm are the best selection in terms of energy consumption or need further tuning. Both algorithms are tested on a real hardware testbed that fully implements a single-cell LTE network (with a real eNodeB and a fully fledged evolved packet core) and a WiFi network using dual-homed single-board computers as sending/receiving nodes.

The rest of the paper is organized as follows. Section 2 reviews the most relevant research works in the literature. The background regarding different MPTCP congestion control algorithms available as Linux kernel implementations is introduced in Section 3. Section 4 outlines the system model. The details of the used experimental setups and procedures are described in Section 5. Section 6 introduces the proposed algorithms. Section 7 presents the experimental results and provides a discussion of the findings. The paper is concluded in Section 8.

## 2. Related Works

Several previous research works address the management of wireless MPTCP connections using an SDN framework. The works propose the usage of SDN to manage MPTCP mainly focusing on routing, controlling the path, and path resilience. For instance, the WRR MPTCP scheduler is used for multipath routing in [20]. A dynamic path control in MPTCP using SDN is proposed by the authors of [21]. In [22], the authors offer a resolution to the bandwidth utilization problem in wireless network sets (WNS) by observing and dynamically controlling the network access by implementing an SDN-based flow management system. The authors of [23] investigate the use of an SDN to manage heterogeneous home networks. They analyze OpenFlow-enabled link switching under normal conditions and link failure situations. Izumi et al., in [24], suggest employing SDN to create MPTCP subflows to reduce the difference in mean delay between them. Other authors consider managing MPTCP connections via SDN in other system models with a similar focus. For instance, the authors of [25,26,27] utilize the SDN-MPTCP framework in naval battlefields, LEO satellites, and V2I communication, respectively.

Several research works address the reduction of MPTCP energy consumption by devising a new scheduling scheme. An energy-aware MPTCP (eMPTCP) model is proposed by [28] and tested on an Android platform to increase energy efficiency, but the bandwidth changes in different scenarios adversely affect the performance gain in eMPTCP. In [29], the authors propose an MPTCP scheduler to save energy by switching to the most efficient network path. They use an MDP with energy models and application tracing to generate schedules. Morawski et al., in [30], create an MPTCP scheduling algorithm for downlink traffic that minimizes energy consumption by distributing loads across multiple interfaces and adjusting channel usage with flow control.

Moreover, the authors in [31] address an MPTCP-based content distribution scheme (eMTCP) that is energy-aware by shifting the traffic to the interface with low energy consumption. Dong et al. [11] propose an energy-saving scheduling system named ES-MPTCP that prioritizes subflows to reduce energy consumption while ensuring network throughput. In [12], the authors propose an energy-efficient multipath scheduler for IoT monitoring systems, which considers both network throughput and energy consumption. In [32], the authors address scheduling in MPTCP using a stochastic optimization approach to maximize the average throughput, reduce congestion, and achieve energy efficiency. They compare the proposed solution using a simulation with the base version of MPTCP and eMPTCP without targeting specific congestion control algorithms. Wu et al. [33,34] propose energy-efficient MPTCP schemes specifically for quality-guaranteed video transmission over heterogeneous wireless networks. They design MPTCP schedulers that consider both the energy consumption of mobile devices and the quality of video transmission by leveraging wireless channel diversity and video frame priority.

A few researchers discuss minimizing MPTCP energy consumption by proposing congestion control schemes different from the Linux kernel implementation ones. For example, the authors of [14] suggest a receiver energy-efficient congestion control method based on MPTCP to allow mobile devices powered by batteries to receive more data than MPTCP while using the same amount of energy. In [13], Zhao et al. propose an energy-aware congestion control mechanism for MPTCP. The proposed algorithm aims to reduce energy consumption while maintaining network performance by shifting traffic to low-delay, lower-energy-consumption paths. Their algorithm’s performance is compared with that of LIA.

Other authors target MPTCP energy consumption analysis and modeling. For instance, the authors of [35] analyze the energy consumption of MPTCP versus standard TCP on mobile devices. They also develop a decision-making process to identify the energy-efficient MPTCP setup for a node streaming with a constant bit rate. An energy consumption model for smartphones with MPTCP or standard TCP is derived in [36] based on real-life measurements by considering four multimedia services such as video streaming, voice over IP, web browsing, and file download. In [37], the authors perform energy consumption measurements of a specific mobile device with handover in different modes of operation. The authors of [38] perform a hardware-based comparative analysis for the energy consumption of three MPTCP congestion control algorithms.

Thus, the previously mentioned research works addressing MPTCP energy consumption mainly aim at changing the current MPTCP Linux kernel implementations by modifying the scheduler [28,29,30,31,32,33,34] or the congestion control algorithm [13,14]. On the other hand, the works combining MPTCP and SDN do not address energy consumption. A comparative summary of the above-mentioned related works is presented in Table 1.

To the best of our knowledge, no research work in the literature experimentally introduce SDN combined with DNN to manage the energy consumption of multiconnectivity wireless nodes running MPTCP, which avoids the need to make any changes in the MPTCP Linux kernel implementation.

## 3. MPTCP Congestion Control Background

The MPTCP protocol contains a scheduler to select the traffic path for each packet and a congestion control algorithm to estimate the amount of data to be sent over each path in order to avoid congestion. The default scheduler of the Linux kernel implementation splits the traffic on each path based on the round-trip time (RTT) experienced over that path. In this section, we provide a basic background of the congestion control algorithms available as a Linux kernel implementation, namely LIA, OLIA, Cubic, BALIA, and wVegas.

### 3.1. Linked Increases Algorithm (LIA)

This algorithm utilizes the RTT and congestion window size of all subflows to determine the congestion window of a subflow, which increases until congestion is detected. When congestion is detected, the transmission rate is reduced to alleviate the issue. The LIA algorithm strikes a balance between the rate of increasing and decreasing the congestion window to ensure a stable transmission rate. It is highly suitable for fluctuating network conditions as it facilitates smooth and stable data transfer while making the most of available resources. LIA utilizes the same algorithms as TCP-Reno, including slow start, fast retransmit, and fast recovery. During the congestion avoidance phase, LIA also employs an additive increase multiplicative decrease (AIMD) algorithm, which increases the congestion window (CW) proportionally to the sum of the congestion windows of all subflows upon receiving an acknowledgment (ACK) on each subflow. The congestion window size increase for MPTCP subflows is calculated using

(1) $$\min(\delta_{1},\delta_{2})$$

where $\delta_{1}=\frac{\alpha }{{\mathrm{CW}}_{\mathrm{total}}}$ and $\delta_{2}=\frac{1}{{\mathrm{CW}}_{i}}$ [39]. The congestion window size for each MPTCP subflow and the combined size of all subflows’ congestion windows are represented by the variables $CW_{i}$ and $CW_{total}$, respectively. The formula calculates the disparity between the increase in congestion window size for the MPTCP subflow and the increase that a typical TCP flow would experience in comparable conditions, where α is given by (2). This approach ensures that MPTCP subflows do not exhibit more aggressive behavior than regular TCP flows in the same scenario [39].

(2) $$\alpha =CW_{total}\times \frac{\max_{i}\left(\frac{C_{i}}{RTT_{i}}\right)}{{\left(\sum_{j}C_{j}\right)}^{2}}$$

where, for any subflow *x*,

$$C_{x}=\frac{CW_{x}}{RTT_{x}}.$$

The terms $C_{i}$ and $C_{j}$ refer to the value of $C_{x}$ for subflows *i* and *j*, respectively. It is assumed that the congestion window size is maintained in terms of packets and the increase in the congestion is per ACK. In (2), the parameter α determines how aggressively MPTCP behaves. This calculation ensures that MPTCP’s total bandwidth is similar to that of a regular TCP flow on the best path available. Additionally, in the event of packet loss, the congestion window size is reduced by $\frac{{\mathrm{CW}}_{i}}{2}$ [39].

### 3.2. Opportunistic Linked Increases Algorithm (OLIA)

OLIA aims to improve performance in changing network conditions. Compared to LIA, the algorithm increases the sending rate more aggressively, leading to the faster use of network resources. It also uses an opportunistic approach to flow control by utilizing the available bandwidth across multiple network paths to achieve higher throughput than regular TCP. Additionally, OLIA has a probing mechanism that allows the sender to evaluate the bandwidth on various paths. This information is then used to adjust the sending rate in real time.

OLIA uses a congestion control algorithm for all subflows and provides TCP-Reno’s response to packet loss. It adopts a slightly modified TCP slow start algorithm to avoid sending traffic over congested paths. OLIA lowers the slow start threshold to one maximum segment size when multiple paths are available, preventing the overloading of any particular path with traffic [39].

(3) $$\frac{\frac{C_{i}}{{RTT}_{i}}}{{\left(\sum_{j}C_{j}\right)}^{2}}+\frac{\lambda_{i}}{{\mathrm{CW}}_{i}}.$$

The first term in (3) incorporates TCP-compatible resource pooling, congestion balancing, and fairness, whereas the second term uses the parameter $\lambda_{i}$ to ensure responsiveness and prevent oscillations. The congestion window increase algorithm is responsive and adaptive to changing network conditions because OLIA adjusts $\lambda_{i}$ based on the availability of subflows with smaller congestion window sizes. This adaptive behavior improves network performance and ensures that resources are used efficiently [39].

### 3.3. Cubic

The Cubic algorithm [40] controls congestion window growth using a gradual and smooth cubic function. It also includes a hybrid slow start mechanism to boost window growth at the start and a fast recovery mechanism to increase the window rapidly after a loss. When implemented in MPTCP, Cubic operates on each subflow independently.

The increase in the congestion window size in Cubic is calculated as

(4) $$\mathrm{CW}=S{\left(T-D\right)}^{3}+CW_{\max}$$

where

(5) $$D=\sqrt[{3}]{\frac{CW_{\max}\cdot \left(1-\gamma \right)}{S}}.$$

Here, γ is the multiplicative decrease factor and *S* is the scaling constant. *T* and $CW_{\max}$ are the time elapsed from the previous window reduction and congestion window size before the last reduction, respectively.

### 3.4. Balanced Linked Adaptation Algorithm (BALIA)

BALIA is a congestion control algorithm that prioritizes both friendly flow behavior and responsiveness to changing network conditions. It dynamically adjusts the sending rate based on the available bandwidth and loss rate, while also balancing the sending rate across multiple subflows. BALIA has proven to be more effective than traditional TCP algorithms, especially in high-bandwidth and high-latency networks.

The BALIA algorithm enhances the effectiveness of TCP’s congestion avoidance algorithm in contemporary network settings that have multiple paths and changing network conditions. BALIA alters both the increase and decrease phases of the AIMD algorithm employed in TCP.

The BALIA algorithm relies solely on the congestion window size and RTT of the subflow paths. Upon receiving an ACK, the increase in congestion window size for subflow *i* is determined using

(6) $$\frac{C_{i}}{{\left(\sum_{j}C_{j}\right)}^{2}}\cdot \left(\frac{\beta_{i}+1}{2}\right)\cdot \left(\frac{\beta_{i}+4}{5}\right).$$

When a congestion event occurs, BALIA decreases the TCP-Reno CW for subflow *i* in the range of [1, 1.5] using (7) as

(7) $$\left(\frac{{\mathrm{CW}}_{i}}{2}\right)\cdot \min\left(\beta_{i},1.5\right)$$

where

(8) $$\beta_{i}=\frac{\max_{j}C_{j}}{C_{i}}.$$

This reduces the size of the congestion window by a factor of 2 multiplied by the minimum of $\beta_{i}$ and 1.5. This ensures that the rate of decreasing the congestion window size is faster for subflows with a larger $\beta_{i}$ value, indicating a higher relative bandwidth share [39]. The value of $\beta_{i}$ is 1 if a single path is used [39].

### 3.5. Weighted Vegas (wVegas)

wVegas [8] is designed to provide a more accurate representation of network conditions. It estimates the RTT for each subflow individually and uses packet marking to detect congestion, instead of relying solely on loss events like traditional TCP algorithms. To adjust the sending rate, it uses a weighted average method, resulting in a more stable and gradual response to changes in network conditions.

## 4. System Model

Since MPTCP is applied with dual or multiconnectivity, where more than one wireless interface is used in data transfer, energy consumption remains a significant issue, especially for devices with limited energy supply (e.g., battery-operated devices).

**Figure 1:**
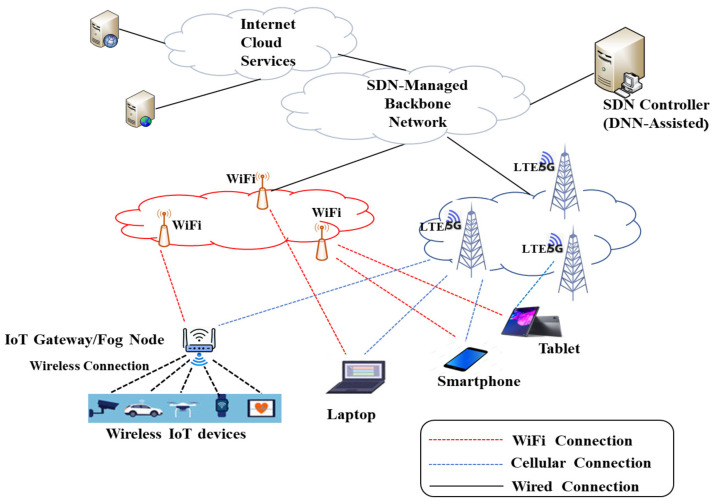
System model.

Consider a scenario where several stationary devices are receiving data traffic from some sending nodes via a communication backbone using MPTCP. The receiving nodes can connect to this backbone through dual connectivity via the simultaneous usage of different radio access technologies (RATs), such as WiFi and LTE/5G. This implies that the receiving nodes are under the overlapped coverage of a WiFi access point (AP) and an eNB or a gNB as shown in Figure 1. The wireless channel of each interface of each node is assumed to be different and affected by the location and the surroundings of the receiving node. This scenario can represent human-based traffic, where users with dual-interface user equipment (UEs) download files or stream data from some server over the Internet. It may also represent dual-interface IoT gateways receiving data from their cloud management entities or servers. This implies that only the receiving nodes are energy-constrained (battery-powered devices), while the sending nodes (e.g., servers) are assumed to be connected to the power grid. Moreover, we assume that all traffic from the sending nodes is routed through SDN-enabled network switches managed by an SDN controller that can steer the traffic of MPTCP subflows before it arrives at the wireless interfaces of each receiving node, and hence it can send the MPTCP traffic over a receiving node’s WiFi interface only, its cellular interface only, or both interfaces. The SDN controller is assumed to be capable of running deep learning artificial intelligence models such as DNNs [41].

## 5. Experimental Setups and Procedures

In this section, the experimental setups used in this research are presented along with the experimental procedures. It is worth noting that due to budget limitations, the cellular configuration of the dual connectivity realization in the testbed is implemented with a reasonable scale using LTE (4G) technology, which can be achieved using communication modems of affordable cost, in contrast to 5G technology.

### 5.1. Setup I Configuration

**Figure 2:**
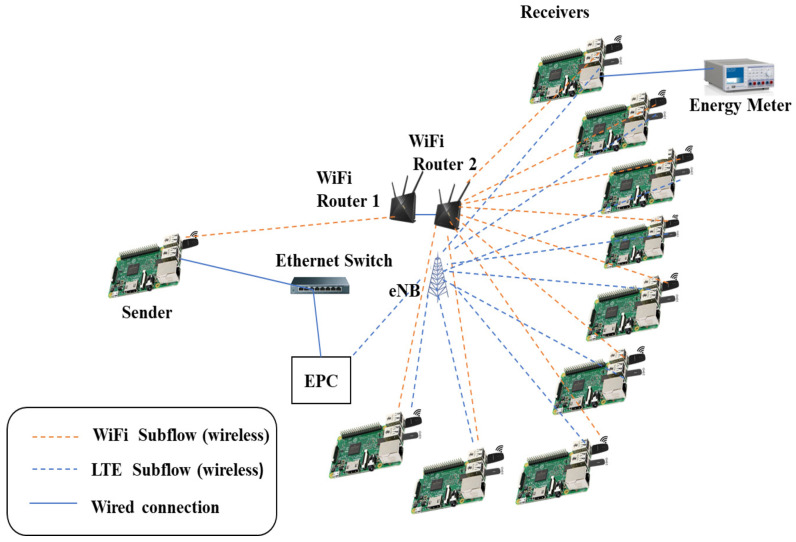
Experimental setup I.

The experimental setup imitates the system model using a testbed based on a real scenario, which enables dual connectivity by transmitting data packets over two wireless networks of different technologies. The wireless nodes employed in the lab experiments are single-board computers (SBCs), which can simultaneously connect to an LTE network via attached LTE universal serial bus (USB) modems and connect to the WiFi network through attached USB WiFi adapters. Due to budget and space limitations, the experiment engages only 10 SBCs: one acts as a sender and the rest are receivers. All SBCs run the Ubuntu Linux server with the Linux kernel implementation of MPTCP, supporting five congestion control algorithms, namely, OLIA, LIA, Cubic, BALIA, and wVegas. The receiving nodes are connected to a WiFi router, which is directly connected to another WiFi router serving the sending node. The two routers run on two different WiFi frequency channels. Moreover, the receiving nodes are connected to a real (not simulated) single-cell LTE network, which consists of a real eNodeB implemented using software-defined radio (SDR) connected to a real evolved packet core (EPC) system implementing the mobility management entity (MME), serving gateway SGW, packet data network gateway (PGW), and home subscriber server (HSS). The LTE network uses the 2.6 GHz band. Therefore, all the LTE modems connect to the network through an isolated RF enclosure, which constrains the locations of the nodes. The sending node has two interfaces. One is connected to a WiFi router, whereas the other is connected to the EPC via an Ethernet switch, as shown in Figure 2.

### 5.2. Setup I Experimental Procedure and Observations

#### 5.2.1. Experiment Procedure

The experimental procedure is performed by transferring a certain amount of data from the sending to the receiving nodes using the MPTCP protocol. The data are generated using the *iperf* traffic generation tool, which is also used to measure the data transfer throughput. The downlink energy consumption is measured during data transfer using an energy meter, as depicted in Figure 2. Each experiment is repeated for around 20 to 30 samples to obtain accurate statistics of the average throughput and energy consumption. The experiment is performed with a varying number of receiving nodes.

#### 5.2.2. Observations

The results of using experimental setup I to measure the energy consumption of dual connectivity receivers running MPTCP with different congestion control algorithms such as OLIA, LIA, BALIA, Cubic, and wVegas are revealed in this section.

**Figure 3:**
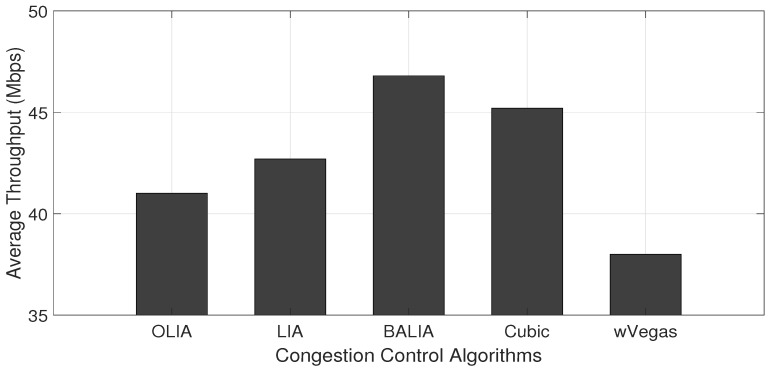
MPTCP data transfer throughput using WiFi and LTE interfaces.

**Figure 4:**
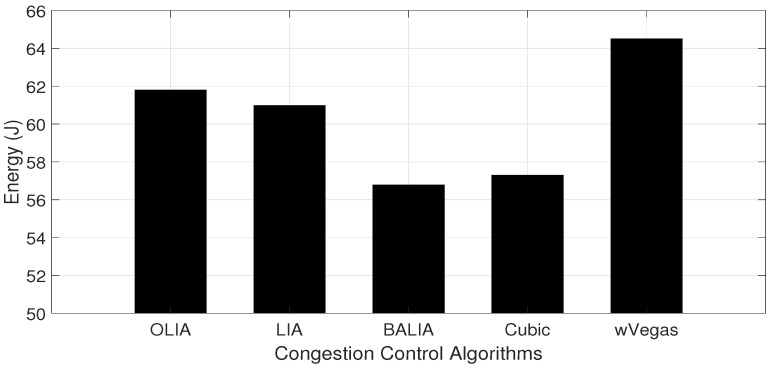
MPTCP energy consumption using WiFi and LTE interfaces.

The energy consumed in the downlink of the receiving nodes when transferring some data volume (60 MB) by the sending node is measured in a scenario where the receiving nodes are varied from one to nine. Figure 3 and Figure 4 show the data transfer throughput and energy consumption obtained with one receiving node for different MPTCP congestion control algorithms, respectively. The figures reveal the direct relation between the throughput and energy consumption. Apparently, the congestion control algorithm capable of transferring with a high throughput achieves lower energy consumption since data transfer happens in a shorter time interval.

Moreover, from Figure 4, it can be noted that BALIA and Cubic consume less downlink energy over dual connectivity MPTCP connections when compared with the other three congestion control algorithms. Thus, in our further experimentation, we use BALIA and Cubic to measure the performance of the proposed algorithms.

**Figure 5:**
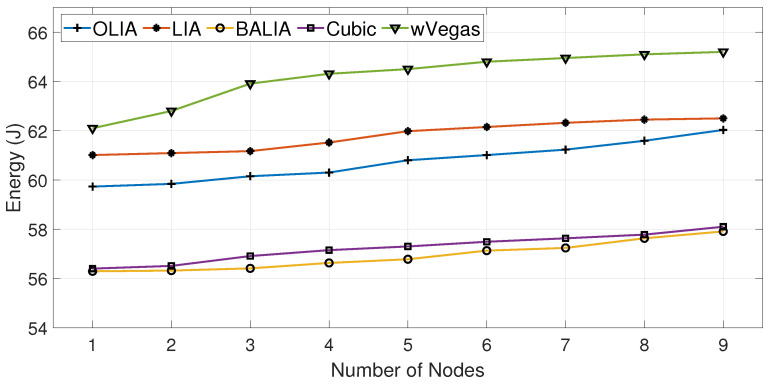
Energy consumption with the number of nodes for different MPTCP congestion control algorithms.

Furthermore, we analyze the energy behavior of different congestion control algorithms while varying the number of receiving nodes, as depicted in Figure 5. The figure shows that the energy consumption increases gradually with the number of nodes, as shown in Figure 5. This is mainly due to the reduction in average throughput as the capacity of the wireless channel is distributed on a larger number of nodes. From the figure, the MPTCP energy value differences while running different congestion control algorithms, such as OLIA, LIA, BALIA, Cubic, and wVegas, are also visible. Again, it can also be noticed that BALIA consumes the least amount of energy, followed by Cubic, which consumes a slightly higher value.

### 5.3. Setup II Configuration

This setup uses a similar configuration to setup I. However, it additionally incorporates an SDN controller and uses a multi-Ethernet interface computer running Open vSwitch (OVS), instead of a normal Ethernet switch. Both WiFi routers (for the sending and receiving nodes) and the EPC system are connected to the OVS switch. The SDN controller is implemented using Ryu for simplicity. It employs the standard OpenFlow protocol [42] to manage the operation of the OVS switch. This includes controlling the switch’s data flow, forwarding rules, and other traffic flows.

### 5.4. Setup II Experiment Procedure

The OVS switch can monitor the incoming packets to each receiving node and record the packet arrival rate, packet size, and transmission duration. By collecting this information, the switch can measure the throughput value of each receiving node and communicate it to the SDN controller. Once the SDN controller receives the throughput measurements from the OVS switch, it utilizes this information to make an informed decision about the interface to be used to transmit the packets.

**Figure 6:**
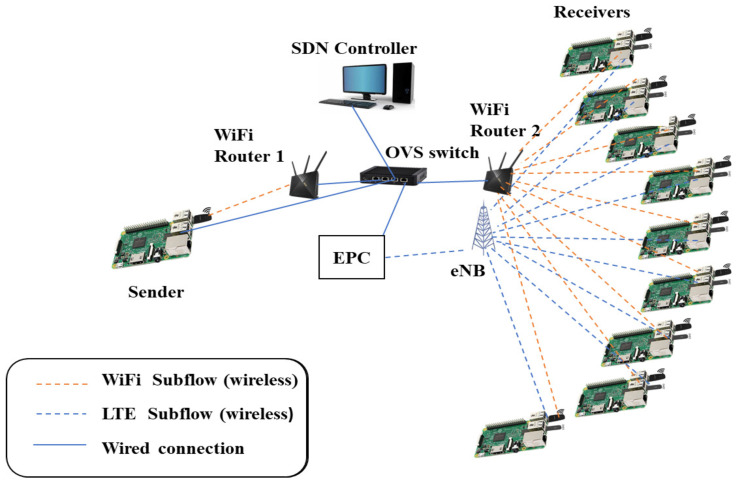
Experimental setup II.

Consequently, the controller manages the data flows by dynamically changing the flow table rules in the switch. For instance, if the data are required to be received only over the WiFi interface of a receiving node, the SDN controller commands the OVS switch to stop forwarding the data of the LTE flow. Similarly, the OVS switch can allow only the LTE flow by dropping the WiFi route from the flow table. The flows through both interfaces are also provided in the flow table of the OVS switch. Ultimately, the entirety of setup II, as shown in Figure 6, automatically provisions the appropriate usage of the interfaces of each node that achieves the least energy consumption by running the algorithms presented in Section 6.

## 6. Proposed Algorithms

The proposed algorithms aim to achieve the lowest overall network energy consumption by sending the data packets through the wireless interface(s) of each node that can achieve this. Thus, the outcome of the proposed algorithms is a combination of interfaces. Here, the term "combination" refers to the mix of interfaces through which the data transfer occurs for each number of nodes. For instance, if three nodes are used, the interface combination that gives the lowest energy consumption may be WiFi for Node 1, LTE for Node 2, both interfaces for Node 3, or the treble (W, L, B). We propose two algorithms. The first selects a combination of interfaces by using SDN (the SDN controller and OVS switch) based on the achieved throughput, whereas the second algorithm performs fine-tuning of the outcome of the first algorithm with the help of neural networks.

The SDN controller runs the developed algorithms as Ryu applications after measuring the achievable channel throughput via the information received from the OVS switch using the OpenFlow protocol. The SDN controller and algorithms are intended to be lightweight. This is easily achieved using the Ryu framework, which also allows seamless integration with machine-learning Python libraries. The SDN controller runs the interface selection algorithm initially and collects the throughputs of the resultant combination of interfaces obtained. Furthermore, these throughputs are given to the trained DNN, and the tuning algorithm runs, as mentioned in the following.

### 6.1. SDN-Based Interface Selection (IS) Algorithm

In this algorithm, the SDN controller probes the achievable throughputs of each interface by sending some traffic via the OVS switch through this interface separately for each node. Indeed, every node has specific achievable throughput values for each interface based on its channel condition.

The IS algorithm compares these achievable throughputs for each interface of each node. The interface that achieves significantly higher throughput than the other is selected to receive the traffic. If the channel conditions of both interfaces lead to slightly different throughput values (within 10%), both interfaces are selected.

While sending the packets from the sender to the receivers, the algorithm checks which of the achievable throughputs of the receivers fall into which of the aforementioned categories. Consequently, the SDN controller alters the flow table accordingly. Thus, the SDN controller manages the data packet forwarding by executing the flow rules on the OVS switch, which forwards the packets to the selected interfaces of the receiving nodes.

Since the achievable throughput controls the amount of time for which an interface is used to receive the transmitted data, it directly affects the energy consumption. MPTCP mainly uses RTT, packet loss, or both to estimate the amount of data to be pushed over each interface and hence the technology used by the wireless interface does not affect the operation of the IS algorithm. Thus, it can be applied to multiconnectivity networks using different technologies, such as 5G, LTE, and WiFi.

In the following steps, we use the first interface and second interface to refer to the two different wireless interfaces used.

- Step 1: Send the probe packets on the first interface of all the nodes and measure the achievable throughput $R_{1}$ of each.
- Step 2: Send the probe packets on the second interface of all the nodes and measure the achievable throughput $R_{2}$ of each.
- Step 3: If a slight difference between the first and second interface throughputs (within 10%) is detected, send the packets through both interfaces.
- Step 4: If the first interface throughput is greater than the second, send the packets through the first interface. Otherwise, send them via the second interface.

**Figure 7:**
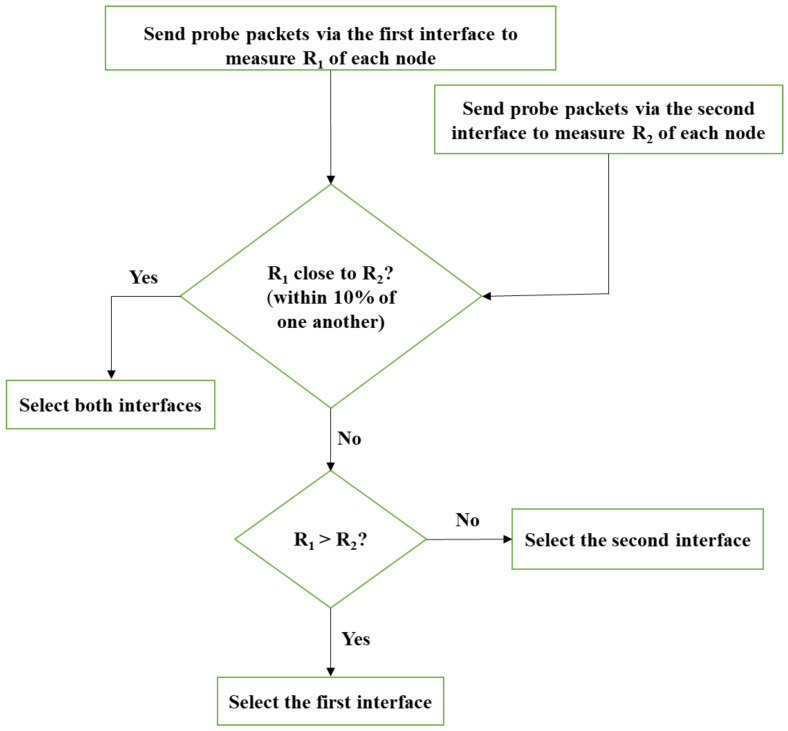
The interface selection (IS) algorithm flowchart.

Figure 7 summarizes the algorithm in a flowchart.

### 6.2. Deep Neural Network-Based Tuning Algorithm

The main objective of this algorithm is to fine-tune the outcome of the IS algorithm based on the energy consumption values provided by a trained DNN.

#### 6.2.1. Dataset Generation and DNN Structure

**Figure 8:**
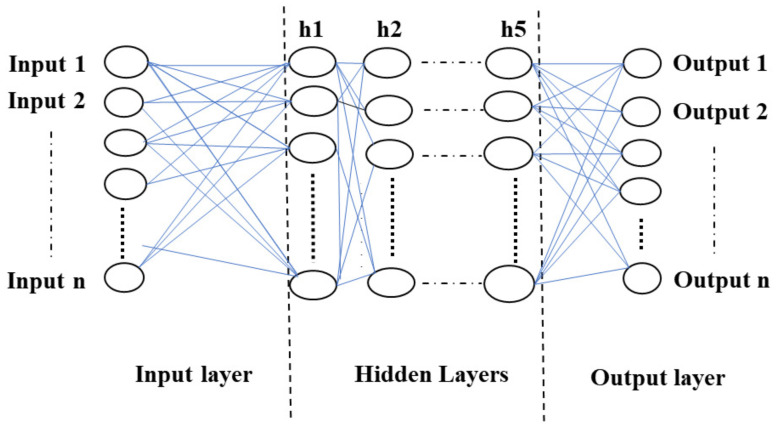
Neural network model.

The dataset for the development of the algorithm is created using experimental setup I. A specific amount (60 MB) of data is transferred from one sender node to a varying number of receiving nodes (from 1 to 9) with different congestion control algorithms, such as OLIA, LIA, BALIA, Cubic, and wVegas. For each case (a certain number of receiving nodes and a congestion control algorithm), the average throughput and corresponding energy consumption are measured when a single interface (WiFi or LTE) is used or both interfaces are. To train the DNN, 30 samples are used for each case. Similarly, 15 samples are used for testing with the DNN. The regressor used is based on a DNN following a multilayer perceptron (MLP) architecture with five hidden layers and 200 neurons in each layer. The ’ReLU’ activation function is used for the hidden layer. The DNN is trained for a large number of iterations on the generated dataset to obtain accurate results. The neural network model is depicted in Figure 8.

#### 6.2.2. The Tuning Algorithm

Since the IS algorithm mainly depends on the achievable throughput to select the interface combination of the receiving nodes, the Tuning algorithm tries to tune or ensure this selection. This is done mainly for the nodes where the achievable throughput of both of their interfaces are slightly different (within 10%). Using the trained DNN and the available information about the achievable throughput of the interfaces of each node, the algorithm calculates the overall network energy consumption based on the outcome of the IS algorithm. After this, the Tuning algorithm revisits the nodes where both interfaces are selected and tries to tune this selection; it then checks the resultant energy consumption using the DNN. The outcome after tuning will only be selected if it achieves lower energy consumption than the outcome of the IS algorithm.

It is worth noting that the Tuning algorithm runs in the SDN controller. It depends on a pre-trained model trained offline on a dataset with measured values that relates the throughput values of the utilized interfaces to the total energy consumption of a user device. Thus, the training time is not included in the operation of the algorithm. Moreover, the DNN training and the algorithm execution do not contribute to the energy consumption of the receiving nodes under study. In addition, the controller is not battery-powered and is supposed to be run by the network provider.

The SDN controller executes the algorithm first by calculating the energy consumption of all nodes from the pre-trained DNN model after starting the transmission using the selected interfaces by the IS algorithm. Next, it simply alters the outcome of the IS algorithm only if both interfaces are selected for a node and uses only the faster interface. After this, it remeasures the throughput values and recalculates the tuned energy consumption for all the nodes using the pre-trained DNN model; it then compares the tuned overall consumption with the overall consumption from the IS algorithm. This makes the complexity of the Tuning algorithm O(n) of the controller processing time needed to calculate the energy consumption of each node using the pre-trained DNN model, where *n* is the number of nodes under the overlapped coverage of an LTE/5G cell and a WiFi AP.

**Figure 9:**
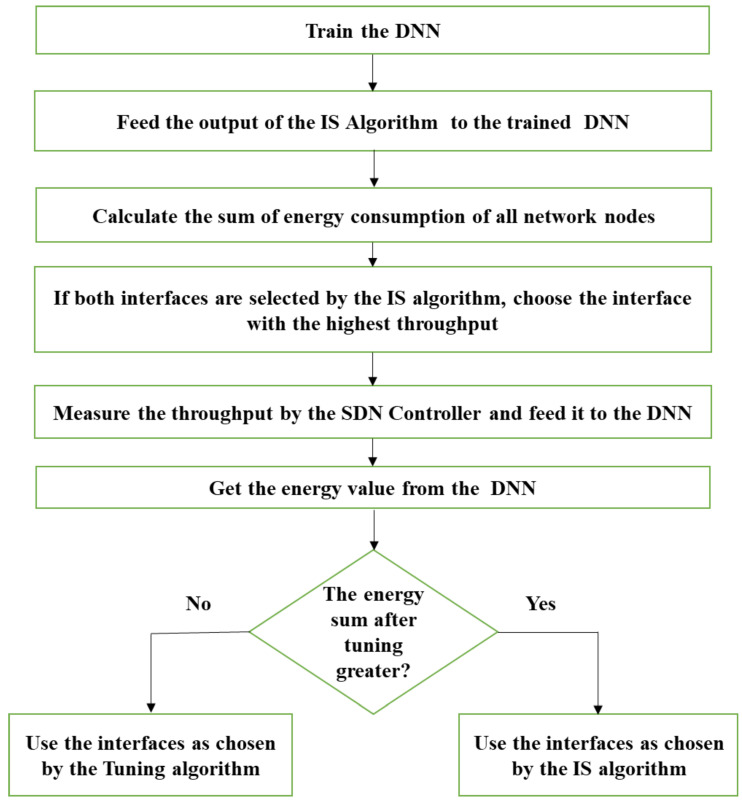
The Tuning algorithm flowchart.

Figure 9 explains the Tuning algorithm in detail.

The following steps compose the Tuning algorithm.

- Step 1: Train the DNN (an offline step).
- Step 2: Feed the output of the IS algorithm for all network nodes to the trained DNN.
- Step 3: Calculate the sum of the energy consumption of all network nodes using the trained DNN based on the measured throughput values.
- Step 4: For the nodes with both interfaces selected by the IS algorithm, select only the interface with the highest achievable throughput.
- Step 5: Measure the throughput by the SDN controller based on the newly tuned selection and feed it to the DNN.
- Step 6: Obtain the energy values of all nodes from the DNN.
- Step 7: Compare the energy values from Step 3 and Step 6; if the energy sum after tuning is greater, use the interfaces as chosen by the IS algorithm for the data transfer. Otherwise, use the interfaces as chosen by the Tuning algorithm.

## 7. Performance Results of Proposed Algorithms

The addition of the SDN in experimental setup II is realized by inserting the OVS switch in the data path between the sending node and the receiving nodes. This allows the SDN controller to execute the proposed algorithms, which run as a Ryu application, select the energy-optimized combination of interfaces, and communicate the selection to the OVS to implement it.

**Figure 10:**
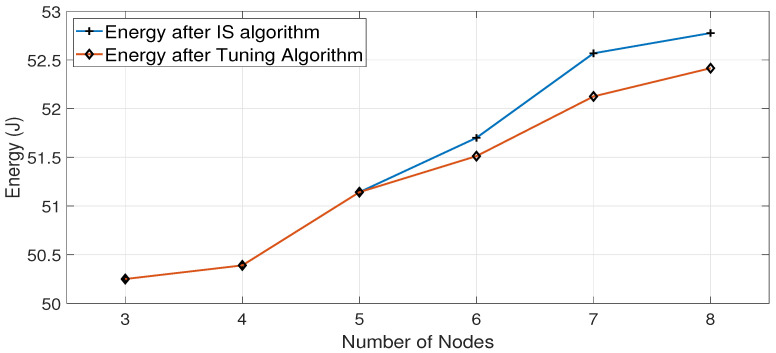
Energy consumption with the number of nodes for Cubic algorithm after applying IS and Tuning algorithms.

**Figure 11:**
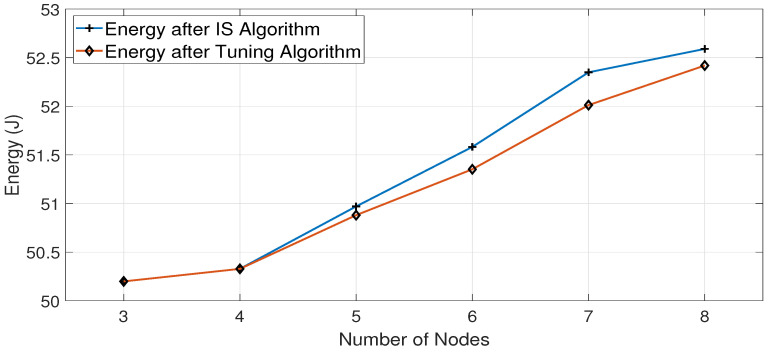
Energy consumption with the number of nodes for BALIA algorithm after applying IS and Tuning algorithms.

Figure 10 and Figure 11 show the energy differences after the Interface Selection algorithm and Tuning algorithm for the Cubic and BALIA congestion control algorithms, respectively.

Figure 10 shows that the Tuning algorithm is successful in reducing energy consumption, especially when the number of nodes increases. This is due to the fact that increasing the number of nodes leads to decreasing the throughput values of both interfaces. Since the throughput is also affected by the channel condition of each interface, this leads to a higher possibility of having nodes with both interfaces achieving close throughput values, which requires the Tuning algorithm to make a fine adjustment to the decision of the IS algorithm. Similarly, in the case of the BALIA algorithm, the Tuning algorithm shows, in general, reduced energy consumption when increasing the number of nodes, as depicted in Figure 11. The reduction in energy consumption after using the DNN-based Tuning algorithm, as presented in Figure 10 and Figure 11, appears to be limited since we use a 60 MB file in the conducted experiments to finish every sample run in a reasonable time. The larger the amount of downloaded data, the greater the energy saving achieved by using the Tuning algorithm per node, which leads to considerable overall energy savings for all battery-powered nodes across the network, given that running the Tuning algorithm does not affect the energy consumption of battery-operated devices.

**Figure 12:**
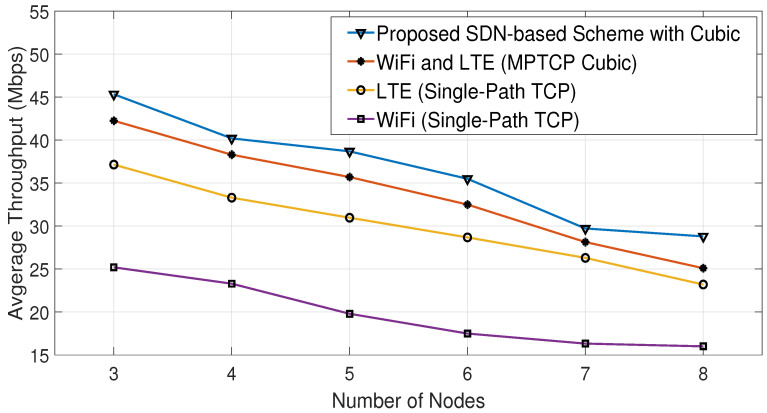
Comparative average throughput using Cubic algorithm.

**Figure 13:**
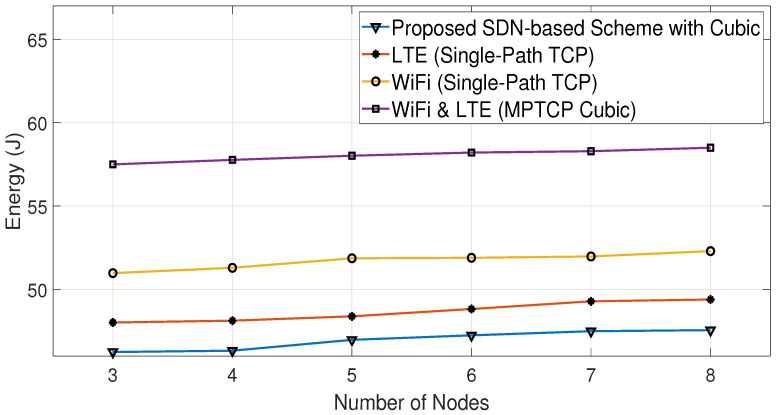
Comparative energy consumption using Cubic algorithm.

**Figure 14:**
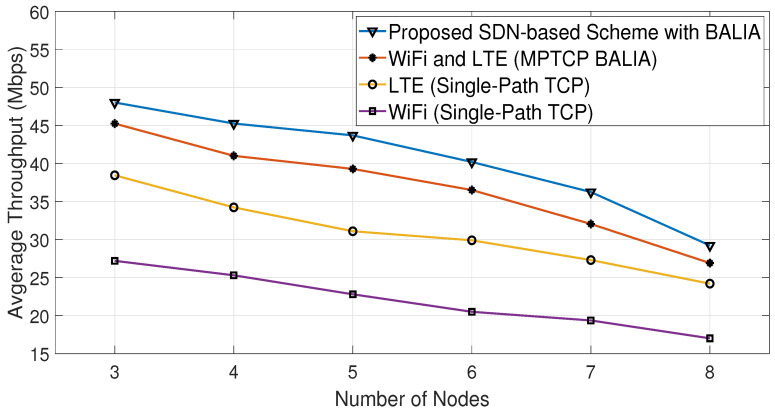
Comparative average throughput using BALIA Algorithm.

**Figure 15:**
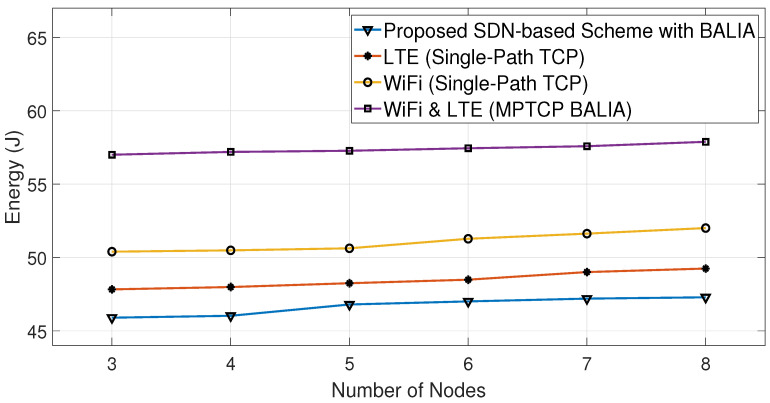
Comparative energy consumption using BALIA algorithm.

Figure 12 shows a comparison of the average throughput of the Cubic algorithm in various scenarios, with and without the proposed SDN-based scheme. These scenarios involve using either single-path TCP (SPTCP) over a single interface (LTE or WiFi) or both interfaces for all nodes. Figure 13 displays the energy consumption results for the same scenarios as Figure 12. It is clear from both figures that using the proposed algorithm leads to increasing throughput and decreasing energy consumption compared with the other studied scenarios. Similarly, Figure 14 and Figure 15 show that the proposed algorithms are able to achieve higher throughput and better energy consumption.

Table 2 and Table 3 summarize the throughput and energy consumption performance comparison, respectively, of the proposed SDN scheme when it is applied with BALIA and Cubic compared with MPTCP BALIA (without the SDN scheme), MPTCP Cubic (without the SDN scheme), LTE (SPTCP), and WiFi (SPTCP).

### Discussion

The proposed algorithms can enhance both the network energy consumption and average throughput because they produce a mixed combination of the scenarios shown in Figure 12, Figure 13, Figure 14 and Figure 15 with the aid of the SDN. This means that certain nodes may only use the WiFi interface, others may only use the LTE interface, and some may use both interfaces based on the channel conditions of their interfaces. This implies that the proposed algorithms indirectly distribute the LTE and WiFi network resources among the receiving nodes, helping them to achieve high average throughput with low energy consumption.

Consequently, based on both algorithms, the SDN controller oversees and makes informed decisions about which interface(s) should be used at each node. This makes the proposed SDN-based scheme able to reduce the energy consumption by 19% compared with MPTCP BALIA or MPTCP Cubic, while achieving, on average, 8% higher throughput than them, as can be observed from Figure 12, Figure 13, Figure 14 and Figure 15 and Table 2 and Table 3. This is because these MPTCP variants activate both interfaces even if one suffers from a poor channel condition, leading to more energy consumption without a significant enhancement in the average throughput.

## 8. Conclusions

This paper addresses the reduction of the energy consumption of multihomed wireless devices using multiconnectivity via heterogeneous radio access technologies to receive MPTCP data packets. We propose an SDN-based approach supported by a DNN to select which wireless interface(s) a network node should use to reduce the overall network energy consumption. The scheme employs two algorithms. One initially selects the wireless interface(s) that should operate on each network node based on the achievable throughput. After this, the other algorithm fine-tunes the selection of the first by applying a DNN to select the interface(s), leading to the lowest overall energy consumption. Both algorithms run at the SDN controller, which implies that no change in the MPTCP implementation, either in the scheduler or the congestion control algorithm, is required. This preserves the features and throughput performance of the currently available MPTCP Linux kernel implementations while retaining operation compatibility.

Two experimental setups mimicking realistic settings are utilized in this research. As the congestion control algorithm used by MPTCP manages the amount of data sent over a wireless interface, affecting its energy consumption, the first setup is used to conduct experimental testing and build a dataset for the energy consumption performance of different MPTCP congestion control algorithms available as Linux kernel implementations, namely LIA, OLIA, Cubic, BALIA, and wVegas. The experimental results show that Cubic and BALIA achieve the lowest energy consumption and the highest throughput among the other MPTCP congestion control algorithms available as Linux kernel implementations. Thus, the second setup tests the proposed scheme algorithms by implementing them on a Ryu-based SDN controller managing an OVS switch via the OpenFlow protocol when using either MPTCP BALIA or Cubic on the sender and receiving nodes.

Extensive experimental results with a variable number of network nodes show that the proposed scheme is efficient in reducing network energy consumption by 19% compared with MPTCP BALIA or MPTCP Cubic while maintaining, on average, 8% higher throughput than these MPTCP variants.

The future directions for this research include predicting the throughput values and energy consumption of fast-moving mobile network nodes using machine learning via the SDN controller. This prediction can be integrated with the proposed algorithms to cope with the fast-changing network topology and node connection status.

## Figures and Tables

**Table 1:** Related works comparison table.

Ref. Number	SDN- Related	Energy- Saving Scheme	Modified MPTCP Component	Summary
[20]	✔	-	-	A WRR MPTCP scheduler controlled by SDN for multipath routing
[21]	✔	-	-	A dynamic path control on MPTCP using SDN
[22]	✔	-	-	A solution for bandwidth utilization problem in WNS using SDN-based flow management
[23]	✔	-	-	A scheme using SDN to manage heterogeneous home networks
[24]	✔	-	-	A scheme using SDN to reduce the delay between MPTCP subflows
[25]	✔	-	-	An SDN-MPTCP framework in naval battle fields
[26]	✔	-	-	An SDN-MPTCP implementation in LEO satellite networks
[27]	✔	-	-	A study on the benefits of SDN and MPTCP in V2I communication
[28]	-	✔	Scheduler	A scheme (eMPTCP) for improving MPTCP energy efficiency
[29]	-	✔	Scheduler	An MPTCP scheduler to choose energy-efficient network path
[30]	-	✔	Scheduler	An MPTCP scheduling algorithm for downlink traffic with energy minimization by load distribution and flow control
[31]	-	✔	Scheduler	An energy-aware MPTCP-based content distribution scheme
[11]	-	✔	Scheduler	A scheduling system (ES-MPTCP) prioritizing subflows for energy minimization
[12]	-	✔	Scheduler	An energy-efficient multipath scheduler for IoT monitoring systems
[32]	-	✔	Scheduler	An energy-efficient MPTCP scheduling scheme using stochastic optimization approach
[33,34]	-	✔	Scheduler	Energy-efficient MPTCP scheduling schemes for video transmission over heterogeneous wireless networks
[14]	-	✔	Congestion Control	A receiver energy-efficient congestion control scheme based on MPTCP
[13]	-	✔	Congestion Control	An energy-aware congestion control mechanism that shifts traffic to low-delay and lower-energy paths
[35]	-	-	-	An energy consumption analysis of MPTCP and power models for streaming data to mobile devices
[36]	-	-	-	An MPTCP energy consumption model for smartphones for different multimedia services
[37]	-	-	-	Energy consumption measurements of a mobile device with handover in different operation modes
[38]	-	-	-	A hardware-based energy consumption comparative analysis of three MPTCP congestion control algorithms
This work	✔	✔	No Modification	Energy consumption management of multiconnectivity devices running MPTCP using SDN and DNN

**Table 2:** Throughput performance comparison (Mbps).

Number of Nodes	Proposed SDN Scheme (with BALIA)	MPTCP BALIA	Proposed SDN Scheme (with Cubic)	MPTCP Cubic	LTE (SPTCP)	WiFi (SPTCP)
3	48	45.25	45.4	42.25	37.8	26.7
4	45.3	41	40.2	38.3	33.7	24.3
5	43.7	39.3	38.7	35.7	31	21.3
6	40.2	36.5	35.5	32.5	29.2	19
7	36.6	32	29.7	28.1	26.8	17.8
8	29.2	26.9	28.8	25.1	23.7	16.5

**Table 3:** Energy consumption performance comparison (Joule).

Number of Nodes	Proposed SDN Scheme (with BALIA)	MPTCP BALIA	Proposed SDN Scheme (with Cubic)	MPTCP Cubic	LTE (SPTCP)	WiFi (SPTCP)
3	45.9	57	46.2	57.5	47.9	50.7
4	46	57.2	46.3	57.8	48.1	50.9
5	46.8	57.3	46.9	58	48.35	51.3
6	47	57.5	47.25	58.2	48.7	51.6
7	47.2	57.6	47.5	58.3	49.2	51.9
8	47.3	57.9	47.5	58.5	49.35	52.2

## Data Availability

No new data were created or analyzed in this study. Data sharing is not applicable to this article.

## References

[1] Madakam S., Lake V., Lake V., Lake V. (2015). Internet of Things (IoT): A literature review. J. Comput. Commun..

[2] (2015). Evolved Universal Terrestrial Radio Access Network (E-UTRAN); S1 general aspects and principles.

[3] (2023). Evolved Universal Terrestrial Radio Access (E-UTRA) and NR; Multi-connectivity.

[4] Paasch C., Bonaventure O. (2014). Multipath tcp. Commun. ACM.

[5] Peng Q., Walid A., Hwang J., Low S.H. (2014). Multipath TCP: Analysis, design, and implementation. IEEE/ACM Trans. Netw..

[6] (2013). Opportunistic Linked-Increases Congestion Control Algorithm for MPTCP.

[7] Le T.A. Improving the performance of multipath congestion control over wireless networks. Proceedings of the 2013 International Conference on Advanced Technologies for Communications (ATC 2013).

[8] Cao Y., Xu M., Fu X. Delay-based congestion control for multipath TCP. Proceedings of the 2012 20th IEEE international conference on network protocols (ICNP).

[9] Rhee I., Xu L., Ha S., Zimmermann A., Eggert L., Scheffenegger R. (2018). CUBIC for Fast Long-Distance Networks.

[10] Kimura B.Y.L., Loureiro A.A.F. (2018). MPTCP linux kernel congestion controls. arXiv.

[11] Dong P., Shen R., Li Y., Nie C., Xie J., Gao K., Zhang L. (2022). An Energy-Saving scheduling algorithm for Multipath TCP in wireless networks. Electronics.

[12] Dong Z., Cao Y., Xiong N., Dong P. (2022). EE-MPTCP: An Energy-Efficient Multipath TCP Scheduler for IoT-based power grid monitoring systems. Electronics.

[13] Zhao J., Liu J., Wang H. On Energy-Efficient Congestion Control for Multipath TCP. Proceedings of the 2017 IEEE 37th International Conference on Distributed Computing Systems (ICDCS).

[14] Wang W., Wang X., Wang D. (2017). Energy efficient congestion control for multipath TCP in heterogeneous networks. IEEE Access.

[15] Xia W., Wen Y., Foh C.H., Niyato D., Xie H. (2014). A survey on software-defined networking. IEEE Commun. Surv. Tutorials.

[16] Fundation O.N. (2012). Software-defined networking: The new norm for networks. ONF White Pap..

[17] Benzekki K., El Fergougui A., Elbelrhiti Elalaoui A. (2016). Software-defined networking (SDN): A survey. Secur. Commun. Netw..

[18] Feamster N., Rexford J., Zegura E. (2014). The road to SDN: An intellectual history of programmable networks. ACM SIGCOMM Comput. Commun. Rev..

[19] Shams R., Abdrabou A. Managing Energy Consumption of Wireless Multipath TCP Connections Using Software-Defined Networking: A Review. Proceedings of the 2021 6th International Conference on Renewable Energy: Generation and Applications (ICREGA).

[20] De Schepper T., Struye J., Zeljković E., Latré S., Famaey J. Software-defined multipath-TCP for smart mobile devices. Proceedings of the 2017 13th International Conference on Network and Service Management (CNSM).

[21] Nam H., Calin D., Schulzrinne H. Towards dynamic MPTCP path control using SDN. Proceedings of the 2016 IEEE NetSoft Conference and Workshops (NetSoft).

[22] Chen K., Xing X., Palash M.R., Liu J., Martin J. Improving wireless network performance under MPTCP based multipath access. Proceedings of the 2018 IEEE 43rd Conference on Local Computer Networks (LCN).

[23] Soetens N., Famaey J., Verstappen M., Latre S. SDN-based management of heterogeneous home networks. Proceedings of the 2015 11th International Conference on Network and Service Management (CNSM).

[24] Izumi K., Ito Y. Proposal of a Method of Reducing Difference of Mean Delay between Paths in MPTCP by SDN. Proceedings of the 2019 IEEE 8th Global Conference on Consumer Electronics (GCCE).

[25] Zhao Q., Du P., Gerla M., Brown A.J., Kim J.H. Software Defined Multi-Path TCP Solution for Mobile Wireless Tactical Networks. Proceedings of the MILCOM 2018—2018 IEEE Military Communications Conference (MILCOM).

[26] Du P., Nazari S., Mena J., Fan R., Gerla M., Gupta R. Multipath TCP in SDN-enabled LEO satellite networks. Proceedings of the MILCOM 2016—2016 IEEE Military Communications Conference.

[27] Singh P.K., Sharma S., Nandi S.K., Nandi S. (2019). Multipath TCP for V2I communication in SDN controlled small cell deployment of smart city. Veh. Commun..

[28] Lim Y.s., Chen Y.C., Nahum E.M., Towsley D., Gibbens R.J. How green is multipath TCP for mobile devices?. Proceedings of the 4th Workshop on All Things Cellular: Operations, Applications, & Challenges.

[29] Pluntke C., Eggert L., Kiukkonen N. Saving mobile device energy with multipath TCP. Proceedings of the Sixth International Workshop on MobiArch.

[30] Morawski M., Ignaciuk P. MPTCP remote peer control for increasing energy efficiency of downlink transmission. Proceedings of the 2016 20th International Conference on System Theory, Control and Computing (ICSTCC).

[31] Chen S., Yuan Z., Muntean G.M. An energy-aware multipath-TCP-based content delivery scheme in heterogeneous wireless networks. Proceedings of the 2013 IEEE Wireless Communications and Networking Conference (WCNC).

[32] Arain Z.A., Qiu X., Zhong L., Wang M., Chen X., Xiong Y., Nahida K., Xu C. (2021). Stochastic Optimization of Multipath TCP for Energy Minimization and Network Stability over Heterogeneous Wireless Network. KSII Trans. Internet Inf. Syst..

[33] Wu J., Tan R., Wang M. (2019). Energy-Efficient Multipath TCP for Quality-Guaranteed Video Over Heterogeneous Wireless Networks. IEEE Trans. Multimed..

[34] Wu J., Cheng B., Wang M., Chen J. (2017). Quality-Aware Energy Optimization in Wireless Video Communication With Multipath TCP. IEEE/ACM Trans. Netw..

[35] Kaup F., Wichtlhuber M., Rado S., Hausheer D. Can multipath TCP save energy? a measuring and modeling study of mptcp energy consumption. Proceedings of the 2015 IEEE 40th Conference on Local Computer Networks (LCN).

[36] Ding T., Yuan Z., Chen S., Muntean G.M. Smartphone energy consumption models for multimedia services using multipath TCP. Proceedings of the 2014 IEEE 11th consumer communications and networking conference (CCNC).

[37] Paasch C., Detal G., Duchene F., Raiciu C., Bonaventure O. Exploring mobile/WiFi handover with multipath TCP. Proceedings of the 2012 ACM SIGCOMM Workshop on Cellular Networks: Operations, Challenges, and Future Design.

[38] Abdrabou A., Prakash M., AlShehi A.S., Ahmed S.E., Darwish M. An experimental study on energy consumption of wireless multipath tcp connections. Proceedings of the 2019 Wireless Telecommunications Symposium (WTS).

[39] Jowkarishasaltaneh F., But J. (2022). An Analysis of MPTCP Congestion Control. Telecom.

[40] Xu L., Ha S., Rhee I., Goel V., Eggert L. (2023). CUBIC for Fast and Long-Distance Networks.

[41] Xu J., Wang J., Qi Q., Sun H., He B. Deep neural networks for application awareness in SDN-based network. Proceedings of the 2018 IEEE 28th International Workshop on Machine Learning for Signal Processing (MLSP).

[42] Bhardwaj S., Panda S.N. (2022). Performance evaluation using Ryu SDN controller in software-defined networking environment. Wirel. Pers. Commun..

